# Profiling the Resident and Infiltrating Monocyte/Macrophages during Rejection following Kidney Transplantation

**DOI:** 10.1155/2020/5746832

**Published:** 2020-09-21

**Authors:** Jie Wang, Peiling Luo, Jingjie Zhao, Junhua Tan, Feifan Huang, Ruiying Ma, Peng Huang, Meiying Huang, Yuming Huang, Qiuju Wei, Liuzhi Wei, Zechen Wang, Lingzhang Meng

**Affiliations:** ^1^Kidney Disease of Internal, Affiliated Hospital of Youjiang Medical University for Nationalities, Baise City, Guangxi Province, China; ^2^Graduate School of Youjiang Medical University for Nationalities, Baise City, Guangxi Province, China; ^3^Life Science and Clinical Research Center, Affiliated Hospital of Youjiang Medical University for Nationalities, Baise City, Guangxi Province, China; ^4^Center for Systemic Inflammation Research (CSIR), School of Preclinical Medicine, Youjiang Medical University for Nationalities, Baise City, Guangxi Province, China; ^5^College of Pharmacy, Youjiang Medical University for Nationalities, Baise City, Guangxi Province, China

## Abstract

Immune tolerance research is essential for kidney transplantation. Other than antibody and T cell-mediated immune rejection, macrophage-mediated innate immunity plays an important role in the onset phase of transplantation rejection. However, due to the complexity of the kidney environment as well as its diversity and low abundance, studies pertaining to monocyte/macrophages in kidney transplantation require further elucidation. In this study, kidney samples taken from healthy human adults and biopsy specimens from patients undergoing rejection following kidney transplantation were analysed and studied. By conducting a single-cell RNA analysis, the type and status of monocyte/macrophages in kidney transplantation were described, in which monocyte/macrophages were observed to form two different subpopulations: resident and infiltrating monocyte/macrophages. Furthermore, previously defined genes were mapped to all monocyte/macrophage types in the kidney and enriched the differential genes of the two main subpopulations using gene expression databases. Considering that various cases of rejection may be of the monocyte/macrophage type, the present data may serve as a reference for studies regarding immune tolerance following kidney transplantation.

## 1. Introduction

A kidney transplant's success largely depends on the degree of immune rejection [1]. Adaptive immunity in kidney transplantation is mainly comprised of T cells and B cells, which, respectively, cause T cell-mediated rejection (TCMR) and antibody-mediated rejection following kidney transplantation (antibody-mediated rejection, ABMR) [2]. Innate immunity, however, primarily involves monocyte/macrophages, which also play an important role in initiating adaptive immunity, and is a prerequisite stage of rejection after kidney transplantation [3].

Myeloid progenitor cells in the bone marrow differentiate into monocytes; then, in the environments of inflammatory reactions and trauma, monocytes migrate and infiltrate the interstitial tissue in order to differentiate into macrophages. However, macrophages can reside in the tissue and renew on their own, crucial for the homeostasis of tissue immunity. These resident macrophages, however, may also be replenished from the circulation in certain conditions [4]. Therefore, macrophage heterogeneity should always be considered in tissue inflammation. In the kidney, earlier studies have shown that the amount of monocyte/macrophage infiltration is closely related to kidney injury, though recent studies have demonstrated that, during the development of acute and chronic kidney disease, mononuclear/macrophage cells have both pathogenic and protective effects according to the type and state of activation. Classically activated macrophages mainly play proinflammatory and profibrotic roles, while alternatively activated macrophages are mainly anti-inflammatory and promote the repair and reconstruction of damaged tissue [5]. In fact, a certain number of resident macrophages exist in normal kidney tissue, which are less understood due to the small number of cells and lack of appropriate means or markers from which to study. The recent development of single-cell sequencing created a breakthrough in studying low abundance cells [6, 7]. Scientists have mapped kidney immune cells throughout the developmental period, from early life to adulthood, and have tried to understand how the kidney's immune system develops and matures [8]. Accordingly, they found that the earliest cells in the developing kidney were monocyte/macrophages, which engulf harmful pathogens and remain in the postnatal stage. However, the roles and characteristics of resident macrophages in the adult kidney, as well as their role during kidney transplantation, require further study.

In this study, the single-cell sequencing data of the kidney was analysed, and the transcriptome of renal resident macrophages was profiled in both healthy and transplant rejection tissue. Here, a certain number of resident monocyte/macrophages were found in normal kidney tissue, which was dramatically reduced in the kidney transplant tissue of immune rejection and replaced by infiltrating monocyte/macrophages. Correlatively, a large number of plasma cells were found in the kidney transplant rejection tissue, where nonactivated B cells disappeared. By analysing the expression profile, infiltrating monocyte/macrophages were observed to be similar to mature M1 macrophages in the common inflammatory response, whereas resident macrophages presented a series of specialized molecules. These molecules were found to be downregulated or were lost monocyte/macrophages from the transplanted rejected kidney. Research on such resident macrophage molecules may provide therapeutic insight into maintaining immune homeostasis, thus assisting in the management of renal transplantation.

## 2. Methods

### 2.1. Single-Cell mRNA Sequencing Analysis

Raw data of healthy kidney and biopsy samples of transplantation rejects were obtained from the Gene Expression Omnibus (GEO) GSE131685 and GSE109564, respectively [9, 10]. R package Seurat [11, 12] was used for data analyses, and count matrices were normalized using the SCTransform pipeline (https://satijalab.org/seurat/v3.1/sctransform_vignette.html). Samples from both healthy and rejection tissue were integrated using reciprocal PCA (https://satijalab.org/seurat/v3.1/integration.htmlA). The integrated dataset was further subjected to PCA, and the initial 15 principal components (which described almost an entire variance in the data, as indicated by elbow plot) were considered for cluster analyses. Unified manifold approximation and projection (UMAP) was used to show clustering with a resolution of 0.25, and violin plots were used to project the expression patterns of individual genes in the cluster.

### 2.2. Functional Enrichment Analysis

Marker genes in different clusters were then annotated for the signaling pathway using the Kyoto Encyclopedia of Genes and Genomes (KEGG) and were classified for biological processes via Gene Ontology (GO) analysis. A *P* value < 0.05 was considered to be statistically significant. A comparison of DEGs was performed by adopting the EnhancedVolcano method, which was illustrated using a volcano graph. DEGs were then compared using a predefined set of genes via Gene Set Enrichment Analysis (GSEA). Molecular Signatures Database (MSigDB) was used as the reference of the DEGs. Furthermore, the R package-ClusterProfiler was used to compare biological processes among the clusters [13]. The top 10 enriched signaling pathways were displayed using dot plots.

## 3. Results

**Figure 1:**
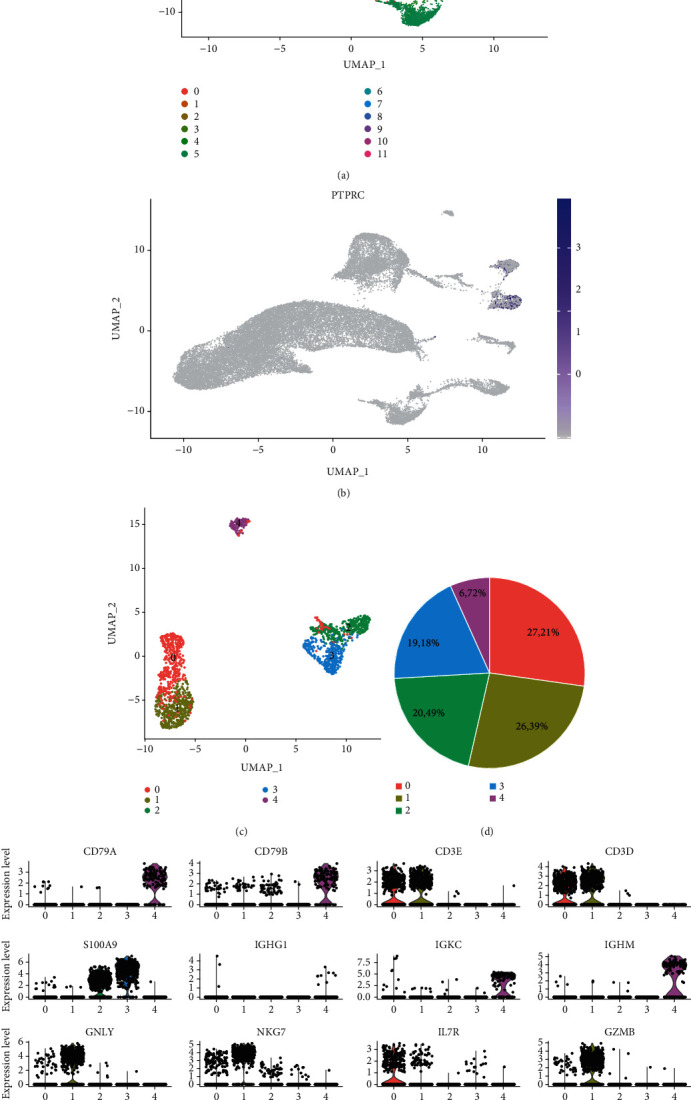
scRNA analysis of leukocytes in the kidney. (a) UMAP plot explored 12 clusters in the kidney tissue at a resolution of 0.25. (b) Gene expression of PTPRC indicated CD45+ leukocytes. (c) CD45+ leukocytes were subclustered into five clusters. (d) Pie plot shows the frequency of each cell type. (e) The subclusters were annotated with known markers for T cells (CD3), monocyte/macrophages (CD14 and MS4A7), and B cells (CD79A/B). Violin plots showed the projection of indicated genes. IGG1 refers to memory B cells or plasma cells. (e) NKT cells were shown with marker genes such as GNLY, NKG7, and GZMB. The IL7R gene refers to conventional T cells.

The data for healthy kidney tissue were acquired from cells isolated in three patients that underwent radical nephrectomy, while the transplant rejection data were taken from one patient with three biopsy samples. Initially, the healthy and rejection data were integrated and corrected in order to eliminate the batch effect. At a resolution of 0.25, 12 cell clusters were generated, in which two leukocyte clusters were verified with PTPRC (CD45 gene) (Figure 1(a)). Subclustering of the leukocytes gave rise to five clusters (Figure 1(b)). Using the known gene markers, the clusters were annotated with (0) T cells (1) ,NKT cells (2, 3) ,monocyte/macrophages, and (4) B cells (Figures 1(c)–1(e)). The healthy data were first assessed (Figures 1(c)–1(e)), and despite the expression of the Igalpha/beta chain (CD79a, b), the B cell population was mainly comprised of IgM (IGHM) but not IgG1 (IGHG1), indicating the lack of plasma cells in healthy tissue (Figures 1(c) and 1(d)). CD3+ conventional T cells and NKT cells may be distinguished by their surface markers and secreted molecules (Figures 1(c) and 1(e)). In monocyte/macrophage populations, cells lack prototypical macrophage gene markers like ADGRE1 (F4/80 gene). Nevertheless, the cells can be sorted into two clusters with CD14 and MS4A7, both myeloid markers (Figure 1(e)), indicating that heterogeneity of kidney monocyte/macrophages already exists in the steady state.

**Figure 2:**
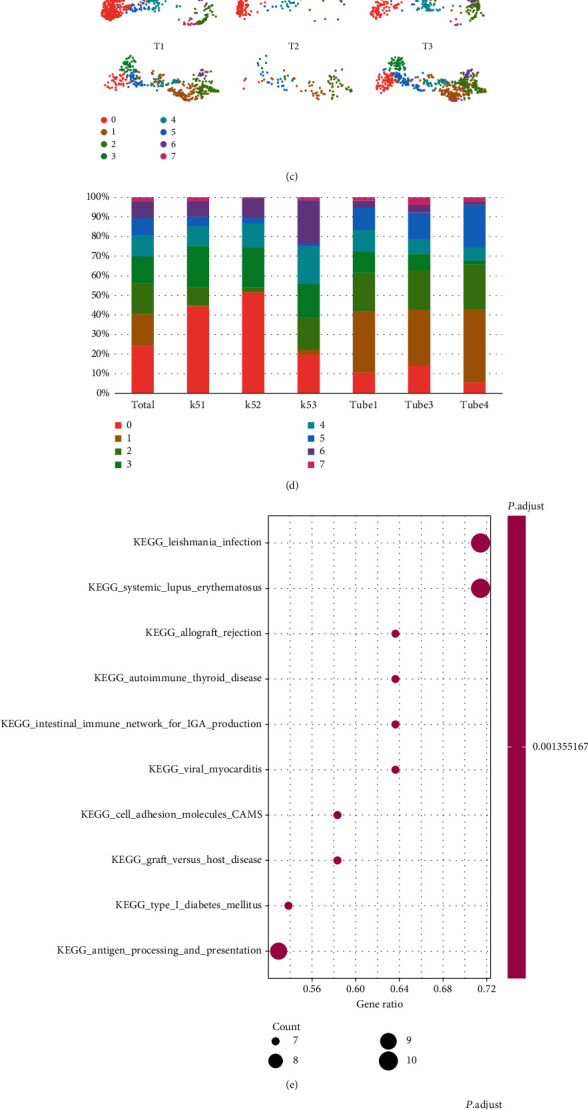
Characterizing the resident and the infiltrating monocyte/macrophage. (a) UMAP plot explored 8 clusters after the removal of B cells. (b) Clusters were annotated with known markers for T cells (3) ,NKT cells (0), and monocyte/macrophage cells (1, 4, and 6) .(c) The cell clusters from the healthy and rejected kidney tissues were compared for individual samples. (d) Bar plot shows the frequency of each cluster of the healthy and rejection samples. The marker genes of clusters 1 (e) and 6 (f) were classified for their involvement of KEGG signaling pathways in clusters 1 and 6.

When comparing the healthy and rejection data, the B cell population was removed so as to obtain a better resolution for the remaining cells. At 0.5 resolution, eight clusters were separated from the rest of the cells (Figure 2(a)). Using known gene markers, the clusters were annotated with (0) NKT cells (3) ,T cells, and five different myeloid cells (Figure 2(b)). Compared to the healthy data, the rejection group demonstrated an evident rise in cluster 1 with a reduction in cluster 6 among all three samples (Figure 2(c)). In order to acquire an overview of clusters 1 and 6, their marker genes were classified via KEGG signaling pathway analysis, where cluster 1 was indeed found to be involved in allograft rejection as well as autoimmune diseases such as lupus and diabetes (Figure 2(c)). In addition, the gene in cluster 1 was found to be relevant to IgA production and antigen presentation, indicating a close relationship to both ABMR and TCMR. However, cluster 6 genes were detected in the phagocytosis pathway (Figure 2(c)).

**Figure 3:**
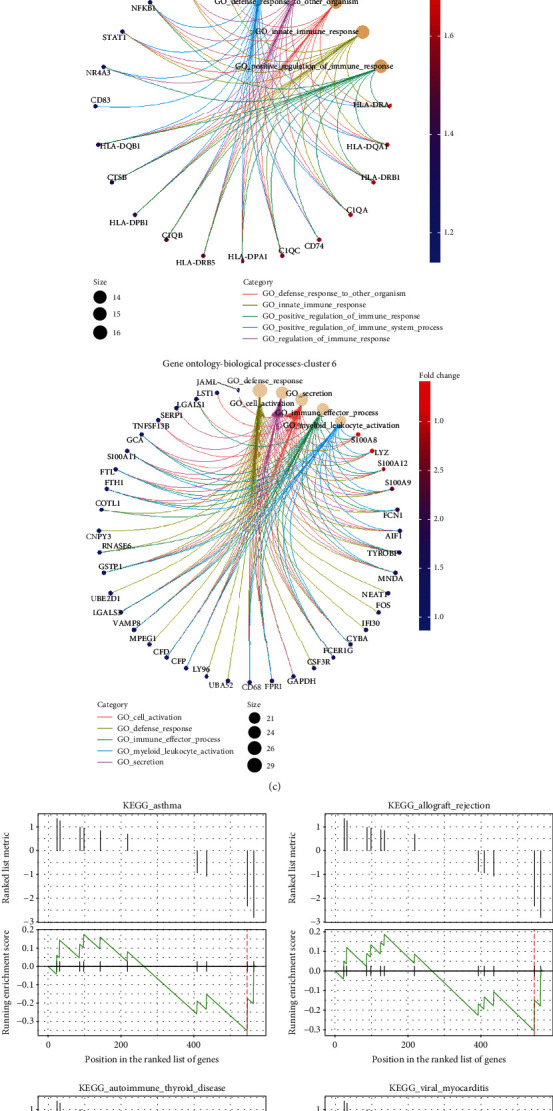
Gene enrichment analysis of the DEGs in the myeloid subsets. (a) Volcano plot demonstrated the statistical significance (*P* value) versus the magnitude of change (fold change) of the DEGs (1 vs. 6). (b) Violin plots showed the percentage and intensity of gene expression by clusters 1 and 6. (c) CNET plots indicated the network of DEGs in clusters 1 and 6. (d) GSEA identified the upregulated or downregulated DEGs in the indicated signaling pathway.

Furthermore, clusters 1 and 6 were compared according to the fold change and *P* value, where cluster 1 was found to possess the gene responsible for antigen presentation and cell activation, as shown in the volcano plot (Figure 3(a)). Moreover, cluster 1 was observed to express most of the markers of the M1 macrophage, whereas cluster 6 expressed the marker of M2 or resident macrophages (Figure 3(b)). To depict the interaction of these genes, a GO enrichment analysis was carried out to illustrate the network of differentially expressed genes. Accordingly, most genes were found to be connected to the immune response; almost all cluster 1 DEGs were involved in the innate immune response (Figure 3(c)). In order to determine which signal is upregulated or downregulated in the DEGs of clusters 1 and 6, a Gene Set Enrichment Analysis (GSEA) was performed, in which the DEGs were compared to the Molecular Signatures Database (MSigDB). The GSEA again oriented the genes to the pathways similar to the KEGG analysis. More importantly, the GSEA confirmed that the genes in cluster 1, not cluster 6, promoted autoimmune disease and allograft rejection (Figure 3(d)).

**Figure 4:**
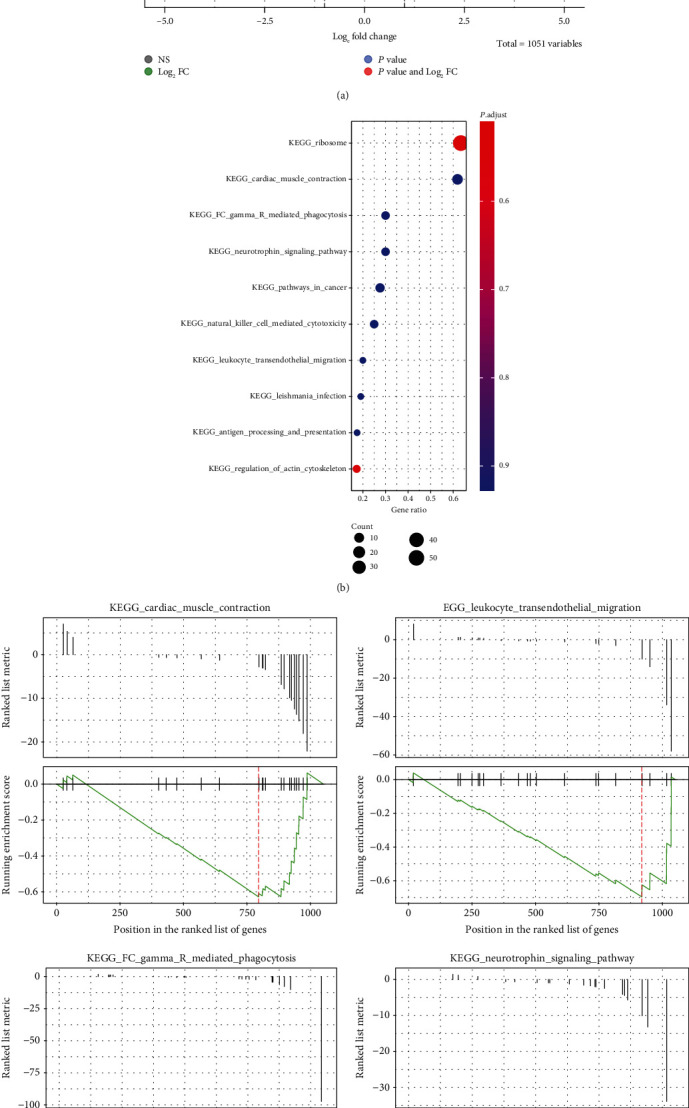
Gene enrichment analysis of the DEGs in both the healthy and rejection kidney. (a) Volcano plot showed the statistical significance (*P* value) versus the magnitude of change (fold change) of the DEGs in cluster 6 (rejection vs. healthy). (b) Dot plot showed the enriched KEGG signaling pathways of the cluster 6 marker genes between the rejection and healthy samples. (c) GSEA analysis identified the upregulated or downregulated DEGs in the indicated signaling pathway (rejection vs. healthy).

Additionally, healthy and rejection samples were compared. As cluster 1 was not detectable in the healthy group, this study focused on the comparison of cluster 6 between the rejection and healthy samples. Here, cluster 6 of the rejection samples was found to upregulate proinflammatory cytokine genes like ILIB while downregulating the expression of CD68, which has been considered a feature of kidney resident macrophages (Figure 4(a)) [14]. To depict the interaction of these genes, a GO enrichment analysis was performed to show the network of differentially expressed genes. The DEGs were mainly found to be involved in leukocyte transendothelial migration and Fcgamma receptor-mediated phagocytosis (Figure 4(b)). The GSEA confirmed that genes in cluster 6 of the healthy group promoted transendothelial migration and phagocytosis, which were lost in the rejection samples (Figure 4(c)).

## 4. Discussion

The major issue of transplantation is remedying rejection following kidney transplantation and prolonging the survival time of the recipient. In this regard, immune tolerance research may contribute to the solution. However, kidney immunity is a complex network, and the phenotype of immune cell is time and space dependent. Therefore, a comprehensive study of cell heterogeneity is required to understand renal immune homeostasis. The present study characterized the genetic expression of renal macrophages at a single-cell resolution and described the changes in resident macrophages as well as their DEGs during transplant rejection. Using enrichment tools, the main markers of both resident and infiltrating monocyte/macrophages involved in kidney rejection were identified and classified.

Due to their high heterogeneity, monocyte/macrophages are known to have different activation states and play different roles. Classically activated M1-type macrophages promote acute kidney injury, glomerulosclerosis, and renal interstitial fibrosis by exerting proinflammatory effects, while alternatively activated M2 macrophages have an anti-inflammatory effect, promoting wound healing, reducing renal inflammatory response and fibrosis, and reducing kidney damage [15]. However, little is known about the role of resident macrophages in healthy kidneys. The present analysis demonstrated that resident macrophages share a certain similarity with M2 macrophages and decreased during the rejection response following kidney transplantation. Other than the known M2 markers, a group of characteristic molecules was also listed, which may serve as a potential target or help in finding new strategies to reduce damage and promote repair in the treatment of kidney disease.

By detecting DEGs featuring the resident macrophage, the relevant signaling pathways of these cells were investigated. In contrast to the M1 macrophage, resident macrophages have little effect in autoimmune disease and allograft rejection. While M1 cells exhibit a strong capacity in the antigen presentation, resident macrophages upregulated relevant genes during phagocytosis, similar to the phenotype of M2 macrophages [16]. Understanding the different biological functions of various monocyte/macrophages may help ascertain their role in renal inflammatory response, injury, and repair. For example, certain immune checkpoint inhibitor therapies often cause kidney damage without the detection of any infiltrating inflammatory cells [17]. One may conceive that such treatments may target and reduce the number of resident macrophages, which is crucial in maintaining the homeostasis of the kidney.

A limitation in the present analysis is that the renal rejection sample was a biopsy from a single patient. Therefore, this analysis does not represent overall chronic renal rejection and may only serve as a useful example. In this regard, additional research is needed in order to describe the immune characteristics of chronic renal rejection. However, the development and application of new technologies, such as single-cell sequencing, transcriptomics, and metabolomics, can propel kidney transplantation immune research to a new level.

## Data Availability

The datasets and code generated or analysed in this study are available from the corresponding author upon reasonable request.
